# The Ecofisio Mobile App for Assessment and Diagnosis Using Ultrasound Imaging for Undergraduate Health Science Students: Multicenter Randomized Controlled Trial

**DOI:** 10.2196/16258

**Published:** 2020-03-10

**Authors:** Mario Lozano-Lozano, Noelia Galiano-Castillo, Carolina Fernández-Lao, Paula Postigo-Martin, Francisco Álvarez-Salvago, Manuel Arroyo-Morales, Irene Cantarero-Villanueva

**Affiliations:** 1 Department of Physical Therapy University of Granada Granada Spain; 2 Sport and Health Joint University Institute Granada Spain; 3 Biohealth Research Institute in Granada Granada Spain

**Keywords:** undergraduate, OSCE, mHealth, teaching and learning strategies

## Abstract

**Background:**

Generation Z is starting to reach college age. They have adopted technology from an early age and have a deep dependence on it; therefore, they have become more drawn to the virtual world. M-learning has experienced huge growth in recent years, both in the medical context and in medical and health sciences education. Ultrasound imaging is an important diagnosis technique in physiotherapy, especially in sports pathology. M-learning systems could be useful tools for improving the comprehension of ultrasound concepts and the acquisition of professional competencies.

**Objective:**

The purpose of this study was to evaluate the efficacy and use of an interactive platform accessible through mobile devices—Ecofisio—using ultrasound imaging for the development of professional competencies in the evaluation and diagnosis of sports pathologies.

**Methods:**

Participants included 110 undergraduate students who were placed into one of two groups of a randomized controlled multicenter study: control group (ie, traditional learning) and experimental group (ie, Ecofisio mobile app). Participants’ theoretical knowledge was assessed using a multiple-choice questionnaire (MCQ); students were also assessed by means of the Objective Structured Clinical Examination (OSCE). Moreover, a satisfaction survey was completed by the students.

**Results:**

The statistical analyses revealed that Ecofisio was effective in most of the processes evaluated when compared with the traditional learning method: all OSCE stations, *P*<.001; MCQ, 43 versus 15 students passed in the Ecofisio and control groups, respectively, *P*<.001. Moreover, the results revealed that the students found the app to be attractive and useful.

**Conclusions:**

The Ecofisio mobile app may be an effective way for physiotherapy students to obtain adequate professional competencies regarding evaluation and diagnosis of sports pathologies.

**Trial Registration:**

ClinicalTrials.gov NCT04138511; https://clinicaltrials.gov/ct2/show/NCT04138511

## Introduction

Generational evolution is nothing new; constant social change poses a challenge for education. During the last several years, concepts referring to subsequent generational learning styles, such as those of Generation X, Generation Y (ie, millennials), or even Generation Z, have been emerging as new ways of understanding new generations [1]. Although most current undergraduate students are within the so-called millennial generation, the next wave of students (ie, Generation Z) is beginning to reach college age [2]. Both groups share many similarities, but the latter has unique characteristics that require new teaching strategies [3]. *Generation Z* is the term used to refer to those born between the mid-90s and the 2000s [4]. This generation has adopted technology from a very early age, creating a deep dependence on it and becoming more drawn to the virtual world [5].

Some studies have shown how these generations absorb and process information in very different ways compared to previous generations [6-8]. These students are characterized as being digital natives and active learners who constantly search for innovation and technology, and who prefer teams, groups, and multitasking [6]. Therefore, they have grown up with several tools that make their learning brain structures different [9]. In this sense, the use of traditional teaching tools is decontextualized [10,11].

In this new teaching-and-learning era, the adaptation of methodologies to new technologies is completely necessary to improve social learning. Educational activity has moved from distance learning to e-learning and, finally, to mobile learning (m-learning), as knowledge has increased exponentially and the demand has escalated [12]. According to Colazzo et al [13], “an educational process can be considered as any learning and teaching activity that is possible through mobile tools or in settings where mobile equipment is available.” M-learning can be understood the same way.

M-learning is surpassing its predecessor (ie, e-learning), since it takes advantage of all its characteristics and overcomes all its disadvantages, making the content much more accessible in time and space [14]. It has become a reality, as proven by many validation studies conducted all over the world [15,16]; it even affects cognitive processes, such as memory. We are becoming symbiotic with our electronic tools, living in an increasingly interconnected society [17], where mobile apps attempt to overcome the problems currently posed by the learning system: the lack of personalized content and appeal to different learning styles, the inability of teachers to apply their true strengths, and the lack of effective reforms at a reasonable cost [18].

There has been an incredible expansion of mobile phone apps in both the medical context [19] and medical and health sciences education. Briz-Ponce et al suggest that more than 37% of health sciences students have used a medical app to improve their learning skills [20]. However, despite the increasing use of m-learning, Po Lau et al highlight the lack of knowledge of m-learning services by public institutions [4]. Even though mobile technology is very well received and health sciences students are well disposed toward its use, its implementation among students has been unsuccessful; this is due to a lack of compliance by the educational systems because m-learning has not been integrated within the standard teaching process [21]. Consequently, as these authors indicate, it is necessary to conduct larger studies to raise awareness of these programs.

There is an increasing number of studies on the use of m-learning in health sciences, such as in surgical skills [15], medicine [21], and nursing [22]. A systematic review in physiotherapy specifically evaluated the effectiveness and user perceptions of online technology for physiotherapy teaching and learning and highlighted its benefits. However, this review did not include any studies that used m-learning as the educational process [23]. Therefore, it is of crucial importance to include this technology as a complement in physical therapy studies.

Our research team has previously conducted some e-learning [24,25] and m-learning studies with physiotherapy students. Our most recent study has shown that palpation skills and ultrasound techniques could be better acquired with a mobile app than by means of traditional learning [26]. In this sense, due to the importance of ultrasound as a diagnostic technique in physiotherapy, especially in sports pathology, m-learning seems to be a useful tool for its study, since it provides the necessary tools for the comprehension of concepts. To our knowledge, there are no studies that propose an m-learning environment to improve the acquisition of professional competencies of sports pathology assessment. Thus, the aim of this study was to evaluate the efficacy and use of an interactive platform accessible through mobile devices using ultrasound imaging for the development of professional competencies in the evaluation and diagnosis of sports pathologies.

## Methods

### Recruitment and Participants

A total of 110 undergraduate physical therapy students took part in this study; students were enrolled at the Health Science Faculty at the University of Granada, Spain, or at the Campus of Tudela, University of Navarra, Spain. These students were in their first or second year of Physical Therapy Fundamentals and Physical Therapy Assessments courses or in their second year of the Physiology of Effort, Physical Exercise, and Health course during the 2014-2015 academic year. Participants’ theoretical knowledge was assessed and they were also assessed by means of the Objective Structured Clinical Examination (OSCE). The participants were recruited via a public announcement at both universities. All students signed an informed consent form to participate in this study.

### Eligibility Criteria

To be eligible for this trial, students needed to meet the following criteria: not having received any previous training in ultrasound imaging and management, having anatomy and biomechanics knowledge, being enrolled in any required subjects, having the basic ability to use a mobile app, and having a mobile phone with Internet access running on the Android operating system.

### Study Design

We conducted a multicenter study, which consisted of a double-blinded, randomized controlled study involving volunteer students earning degrees in physical therapy from two public Spanish universities. The study was registered at ClinicalTrials.gov (NCT04138511). We used a computer program to randomly assign a number to each student in both universities. These numbers were provided in numbered, opaque envelopes by an external study member to ensure that the study member responsible for outcome assessments was blinded to intervention and control group assignment. The envelopes were opened after a baseline assessment. The assessment evaluator was blinded to both the assessment and the outcomes. There were 55 participants in the experimental group and 55 in the control group. The students were also blinded to the group to which they were assigned.

After students received theoretical and practical lessons about ultrasound skills in sports pathology areas, the role that the Ecofisio mobile app played was assessed in a traditional way and by means of the OSCE. The OSCE allows us to measure ultrasound competence as expressed in specific observable behaviors. This study was approved by the *Unidad de Calidad, Innovación y Prospectiva—*a quality and innovation body—from the University of Granada, Spain (PID 14-56), and was conducted in accordance with the Declaration of Helsinki [27,28].

The theoretical and practical lessons were conducted by three professors and three teaching fellows; the teacher-to-student ratio was 1:6-8. The same lessons were taught in both universities by the same professors.

There were a total of six contact sessions on site; this was followed by self-study time focused on the theoretical and practical learning of ultrasound imaging procedures, on different areas of the body, regarding sports pathologies. Each study group—Ecofisio intervention group and control group—attended 2 hours of theoretical lessons and 4 hours of practical lessons. The ultrasound imaging sessions were developed following a previously reported methodology [29] and the diagnosis of sports injuries. All participants used the same ultrasound device model—MyLab 25 (Esaote Medical Systems)—with a 12 MHz linear probe. Finally, there was a self-study period, where the Ecofisio group used the mobile app and the control group used traditional study models (eg, books and journal papers).

Both groups had 2 weeks to study after the on-site lessons. To prevent the control group from accessing information related to the mobile app, they were assessed before the Ecofisio group. The Ecofisio mobile app is focused on sports pathologies and contains relevant written and digital information about ultrasound imaging and management, as well as diagnosis of sport injuries. For each anatomical structure, there exists a theoretical description, a drawing with the anatomical description, an image with the specific placement of the ultrasound probe, an ultrasound slice, a diagram of the ultrasound image, and a video of the manual palpation procedure.

### Outcome Measures

All variables were measured among both study groups after their self-study periods.

#### Objective Structured Clinical Examination

An OSCE [30,31] was used to measure participants’ hands-on skills in terms of ultrasound management. It is a competency-based evaluation [32] consisting of one station with five specific components: positioning of the patient, positioning of the ultrasound probe, orientation (ie, angle) of the ultrasound probe, handling of the ultrasound probe, and image adjustment. Before starting each component, the participants received accurate information about the task to perform. The examiner used a 5-point Likert scale, ranging from 0 to 4, to grade the specific efficiency of each component. The examiner also registered the required time (seconds) to identify the lesion. During each component, no comments were allowed between the students, the examiners, and the patients. The blinded examiner was an expert in ultrasound management with at least 5 years of experience, and the patient suffered a lesion that students had to locate. There were different clinical cases about different sports injuries.

#### Multiple-Choice Questionnaire

An evaluation of students’ theoretical knowledge was conducted using a multiple-choice questionnaire of 20 questions, where a maximum score of 10 points could be obtained.

#### Satisfaction Survey

Satisfaction scores related to the interventions were assessed among both groups through a specific 5-point Likert questionnaire, ranging from 1 (*disagree*) to 5 (*strongly agree*), which had been used in similar contexts previously [24-26]. Furthermore, the Ecofisio group completed another satisfaction questionnaire about the usage of the mobile app, ranging from 0 (*totally unsatisfied*) to 10 (*totally satisfied*).

### Sample Size Calculation

It would require a power of 90% to detect a significant mean difference of 3.5 (SD 3) points in the palpation assessment via the OSCE, assuming a type 1 error (alpha) of 5% and a type 2 error (beta) of 10%. Considering a dropout rate of 20%, we decided to enroll 55 subjects per group. Before the on-campus sessions, an independent researcher randomly assigned each participant to a group using Epidat 3.1 software (Xeral de Saúde Pública).

### Statistical Analysis

Descriptive analyses were obtained for all randomized participants. The Kolmogorov-Smirnov test was applied to test the hypothesis of normality for all variables. We calculated Student *t* tests for variables with normal distribution and Mann-Whitney U tests for the rest as nonparametric tests. The statistical significance was determined by *P*<.05, and all analyses were conducted using IBM SPSS Statistics for Windows, version 22.0 (IBM Corp).

## Results

**Figure 1:**
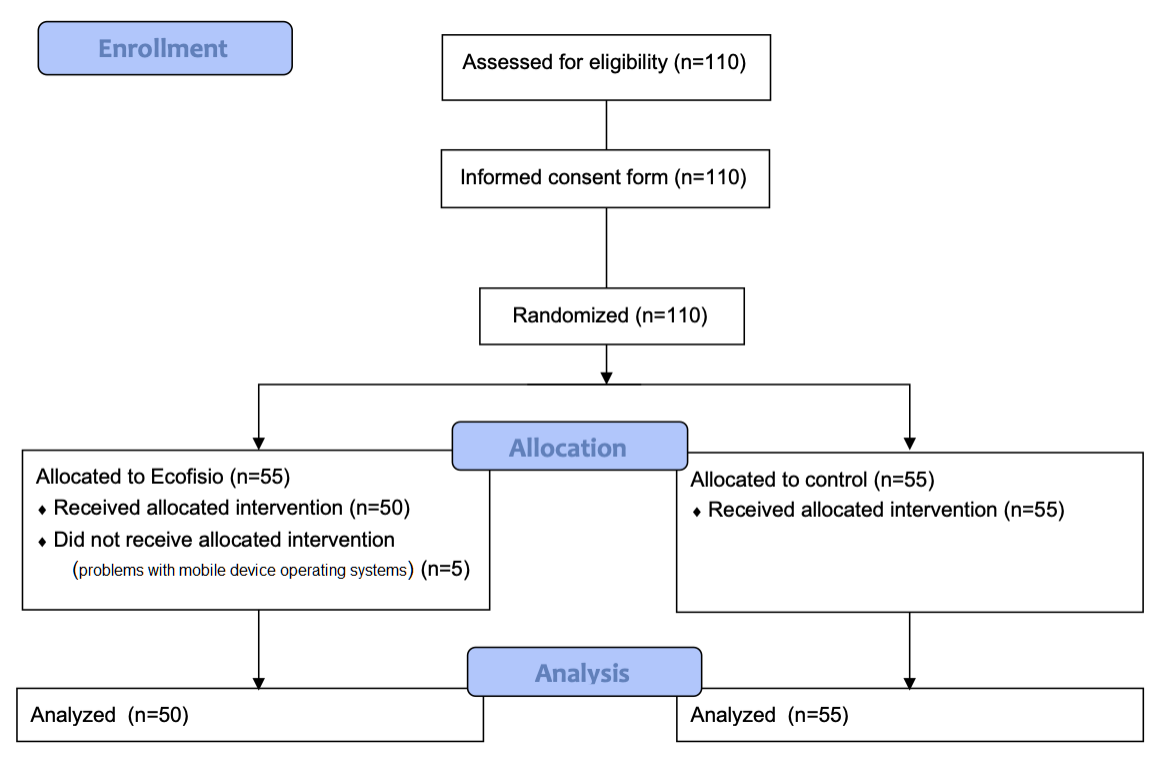
Consolidated Standards Of Reporting Trials (CONSORT) flow diagram of the study.

The final sample was comprised of 50 students in the Ecofisio group and 55 students in the control group, with mean ages of 19.8 years (SD 2.1) and 19.7 years (SD 6.0), respectively. At the beginning of the study, there were no group differences in study variables such as gender or previous experience (see Table 1). There were 5 dropouts (5/55, 9%) in the Ecofisio group due to problems with the operating system versions of their mobile devices (see Figure 1).

**Table 1:** Characteristics of groups.

Characteristic		Ecofisio group (N=50)	Control group (N=55)
Age (years), mean (SD)		19.8 (2.1)	19.7 (6.0)
**Gender, n (%)**			
	Female	30 (60)	32 (58)
	Male	20 (40)	23 (42)
**Experience with ultrasound, n (%)**			
	Yes	0 (0)	0 (0)
	No	50 (100)	55 (100)
**Experience with sports injuries diagnosis, n (%)**			
	Yes	0 (0)	0 (0)
	No	50 (100)	55 (100)
**Experience with sports injuries diagnosis with ultrasound, n (%)**			
	Yes	1 (2)	4 (7)
	No	49 (98)	51 (93)

We found a significant difference in the theoretical exam score (*P*<.001) in favor of the Ecofisio group: mean 7.3 (SD 1.5) points. The results also indicated significant differences in all components assessed during the OSCE stations (*P*<.001 for all) (see Table 2). The Ecofisio group significantly improved in the positioning of the patient (mean score 3.9 [SD 0.3]) and ultrasound probe (mean score 3.9 [SD 0.3]) compared with the control group (mean score 2.3 [SD 1.4] and 1.6 [SD 1.5], respectively). The Ecofisio group also reported higher marks than the control group in the following tasks: orientation of the ultrasound probe (mean score 3.8 [SD 0.6] vs 1.8 [SD 1.7]), handling of the ultrasound probe (mean score 3.3 [SD 0.8] vs 0.7 [SD 0.9]), and image adjustment (mean score 3.0 [SD 1.4] vs 1.6 [SD 1.5]). Finally, the Ecofisio group took significantly more time (*P*<.001) to identify the lesion (mean score 60.2 [SD 25.9]) than did the control group (mean score 45.3 [SD 18.3]). We found significant differences between groups in terms of passing the exam in favor of the Ecofisio group (43/50, 86%, vs 15/55, 27%, *P*<.001) (see Table 2).

Regarding the satisfaction results, we only found significant differences between groups for two items. The Ecofisio group showed a higher satisfaction level than the control group for the following items: *The teacher was competent* (mean score 4.8 [SD 0.4] vs 4.5 [SD 0.5], *P*=.009) and *I believe that the training is applicable* (mean score 3.3 [SD 0.8] vs 3.0 [SD 0.9], *P*=.04) (see Table 3). For the rest of the items, there were no significant differences between groups: *The subject was interesting* (*P*=.08) and *I am satisfied with the training* (*P*=.98) (see Table 4).

**Table 2:** Differences between groups in data obtained from the Objective Structured Clinical Examination (OSCE) stations 1 and 2.

Station, specific components		Ecofisio group (N=50)	Control group (N=55)	*P* value
**1, mean (SD)**				
	Total theoretical knowledge (maximum 10 points)	7.3 (1.5)	6.3 (1.5)	<.001^a^
**2, mean (SD)**				
	Positioning of patient (maximum 4 points)	3.9 (0.3)	2.3 (1.4)	<.001^a^
	Positioning of ultrasound probe (maximum 4 points)	3.9 (0.3)	1.6 (1.5)	<.001^a^
	Orientation (ie, angle) of ultrasound probe (maximum 4 points)	3.8 (0.6)	1.8 (1.7)	<.001^a^
	Handling of ultrasound probe (maximum 4 points)	3.3 (0.8)	0.7 (0.9)	<.001^a^
	Image adjustment (maximum 4 points)	3.0 (1.4)	1.6 (1.5)	<.001^a^
	Time to identify the lesion (seconds)	60.2 (25.9)	45.3 (18.3)	<.001^b^
**2, total, n (%)**				
	Suitable	43 (86)	15 (27)	<.001^c^
	Not suitable	12 (14)	35 (73)	<.001^c^

^a^Mann-Whitney U test.

^b^One-way analysis of variance (ANOVA).

^c^Chi-square test.

**Table 3:** Differences between groups regarding training satisfaction.

Item	Ecofisio group (N=50), mean (SD)	Control group (N=55), mean (SD)	*P* value
The teacher was competent (maximum 5 points)	4.8 (0.4)	4.5 (0.5)	.009^a^
The subject was interesting (maximum 5 points)	4.3 (0.5)	4.4 (0.6)	.08^a^
I am satisfied with the training (maximum 5 points)	3.6 (0.7)	3.5 (0.7)	.98
I believe that the training is applicable (maximum 5 points)	3.3 (0.8)	3.0 (0.9)	.04^a^

^a^Mann-Whitney U test.

**Table 4:** Descriptive data related to the Ecofisio group’s satisfaction.

Item			Ecofisio group (N=50), n (%)
**Understandable information**			
	Very clear	40 (80)	
	Not very clear	10 (20)	
	Not clear	N/A^a^	
**Scientific quality of information (good quality)**			
	Yes	46 (92)	
	Not quite	4 (8)	
	No	N/A	
**Clear presentation of contents**			
	Yes	37 (74)	
	In some cases	13 (26)	
	No	N/A	
**Overall impression of the app**			
	Very good	7 (14)	
	Good	43 (86)	
	Not very good	N/A	
	Bad	N/A	
**Evaluation of the app from 0 to 10**			
	10	1 (2)	
	9	11 (22)	
	8	28 (56)	
	7	10 (20)	
	6	N/A	
	5	N/A	
	4	N/A	
	3	N/A	
	2	N/A	
	1	N/A	
	0	N/A	

^a^Not applicable.

## Discussion

### Principal Findings

This work presents the results obtained on the use and efficacy of an interactive platform accessible through mobile devices using ultrasound imaging for the development of professional competencies in the evaluation and diagnosis of sports injuries. The mobile app has demonstrated its effectiveness in the majority of the processes evaluated when compared with the traditional learning method. Moreover, the results revealed that the app is attractive and useful from the point of view of the students.

The findings presented are in line with previous work developed by our research group, where different technological tools were used to acquire professional competencies among physiotherapy students [24-26]. This study improves and adds importance to the ultrasound imaging competencies for the assessment and follow-up in sports injuries, which has great impact on the development of the professional activity of physiotherapists. In the case of the use of a mobile app, this study resulted in an improvement in all the components of the ultrasound assessment, being broader than those presented previously by Fernández-Lao et al [26]; that study, which also used a mobile app, showed no differences between groups in the items *orientation of the ultrasound probe* and *image adjustment*. This may be due to the fact that students had more motivation to correctly diagnose the sports injuries than to improve on other settings presented in the previous work. On the other hand, in our opinion, more experienced teachers could have also had a positive influence on the way information was provided to students.

A surprising result was that there were significant differences between groups in the time required to identify the lesion. The identification time was almost 20 seconds lower for the control group when compared with the Ecofisio group. This finding was also described in our previous work [26]. We hypothesized that the students in the m-learning group took longer to reach the final result because they dedicated more time to finding a clearer image of the lesion. It is also possible that the control group had finished before the m-learning group because they did not find a clear image of the lesion [33]. Nevertheless, decreasing the time to identify the lesion was not an objective of our study and, therefore, the students were not encouraged to complete the assessment in a short time.

According to the results of the postprogram survey on training satisfaction, nearly all the scores were better in the Ecofisio group than in the control group. We think that the students in the group with access to the app felt a greater motivation for the project in general, so they felt more satisfied with the experience; this fact was also stated in all our previously published works [24-26]. This is in accordance with results from Hill et al, where the students preferred new technologies over traditional learning processes [6]. Another previous study developed in the area of physiotherapy, which used a 3D mobile app, also showed a very good level of acceptance among students of anatomy and manual therapy [34]. In our study, the only item in which both groups presented very similar results was *I am satisfied with the training*. It is possible that all the students who were enrolled in the project were motivated because the project was conducted during the course and because of their very active participation in any activity that was proposed. Furthermore, their regular teacher taught the lessons, in which students were encouraged to consider the mobile app as a study complement.

At the end of the project, the students in the Ecofisio group were asked about their level of satisfaction with the app; the results were quite good for all of the items evaluated. A total of 80% of the students gave scores of 8 or more on a 0-10-point rating scale about their overall satisfaction level with the app, similar to our previous studies [24-26]. Fernández-Lao et al [26] reported an average score of 8.2 on the global satisfaction level with their mobile app as a learning complement of palpation and ultrasound imaging skills of the shoulder. This fact demonstrates that, regardless of the subject studied with mobile apps, these tools are useful for students and their reported acceptance levels are very good. This fact highlights the interest and familiarization of *Generation Z* students with new technologies [35,36].

### Limitations

Finally, we have to recognize some limitations of this research. Our study results with this mobile app should be supported in different countries and with different languages in order to extrapolate the results to other populations. On the other hand, we are aware of the fact that conducting the project during the course sessions may have prompted extra motivation among the students and that ultrasound imaging was an attractive tool for them to use; therefore, the results were quite good for both study groups. In this sense, it would be interesting to develop an independent study with the mobile app in other settings. Finally, there was no follow-up period with which to evaluate the sustainability of the benefits of this interactive platform. Nevertheless, this study also presents a number of strengths. This project was carried out in two different universities in Spain with an important sample of students; therefore, the results could be generalized to physiotherapy students. To our knowledge, this is one of the few studies that has been developed in the area of physiotherapy, showing the use of new technologies to complement traditional learning. In fact, we believe this is the first m-learning system designed to improve the acquisition of professional competencies in the assessment of sports pathologies. In addition, this m-learning system could be implemented in different areas of knowledge, such as sports medicine or sports sciences.

### Conclusions

Ecofisio is an interactive platform accessible through mobile devices, which uses ultrasound imaging. It is an effective way to develop professional competencies in the evaluation and diagnosis of sports pathologies in physiotherapy students, in a way that students find satisfying. Similar experiences could be implemented in different areas of knowledge with good results.

## Appendix

Multimedia Appendix 1 CONSORT-eHEALTH checklist (V 1.6.1).

## Abbreviations

m-learning: mobile learning
OSCE: Objective Structured Clinical Examination
